# Internal Use of Water in Typhoid Fever

**Published:** 1891-08

**Authors:** 


INTERNAL USE OF WATER IN TYPHOID FEVER.—Beverly Robinson, M. D., believes that free diuresis should be obtained in order to promote recovery in typhoid fever cases; that free drinking of water promotes that effect. Refers to the experience of Debove, Meigs, Beaumetz and Cantania as factors in inducing him to try the treatment in his practice and at St. Luke's Hospital. His experience corroborates the others that in the free use of water internally that free diuresis is established, elimination promoted, temperature lowered, and course of disease favorably modified. He had no difficulty in giving four to six ounces of water every two hours in addition to three or four pints of milk that was taken daily. This amount of fluid caused no trouble, patients taking it without a murmur. Urine increased, temperature lowered. He does not say it will be as beneficial as Brand's method of cold baths, but considers it nature's prescription, and can do no harm.—*Medical Record, July, 1891.*

